# Clinical Utility of Contrast-Enhanced Ultrasound (CEUS) in Urology: A Multisystem Review

**DOI:** 10.7759/cureus.94690

**Published:** 2025-10-15

**Authors:** Haadia Safdar, Mecaelan Sardar, Niharika Tekchandani, Ahsan Iftikhar, Haider Iftikhar, Faisal Ghumman, Javed Burki

**Affiliations:** 1 Urology, Medway NHS Foundation Trust, Gillingham, GBR; 2 Institute of Medical Sciences, Canterbury Christ Church University, Canterbury, GBR; 3 Medical Education and Simulation, Medway NHS Foundation Trust, Gillingham, GBR; 4 Hospital Medicine, Medway NHS Foundation Trust, Gillingham, GBR; 5 Medicine, City St. Georges University of London, London, GBR; 6 Hospital Medicine, Army Medical College, Rawalpindi, Rawalpindi, PAK

**Keywords:** article review, ceus, contrast-enhanced ultrasound, urological pathology, uro-radiology

## Abstract

Contrast-enhanced ultrasound (CEUS) is a non-invasive, versatile imaging technique with an expanding spectrum of uses across a wide variety of urological disorders. By utilizing microbubble-based contrast agents confined to the intravascular space, CEUS provides real-time, high-resolution assessment of tissue vascularity without ionizing radiation or nephrotoxic contrast. This review presents an updated overview of CEUS applications in urology, including renal mass characterization, staging of bladder cancer, assessment of scrotal trauma, fertility interventions such as microdissection testicular sperm extraction (M-TESE), and adjunctive prostate cancer imaging. Despite multiple advantages, the more widespread use of CEUS is limited by its limited availability and the absence of standardized imaging protocols. As evidence becomes increasingly available and consensus guidelines emerge, CEUS will take its place as a fundamental tool in the diagnostic armamentarium of modern urological practice.

## Introduction and background

Contrast-enhanced ultrasound (CEUS) is an advanced imaging method that enhances standard ultrasound by incorporating intravenous administration of specialized ultrasound contrast agents (UCAs), offering the benefits of a non-invasive technique alongside improved diagnostic precision through contrast enhancement [1].

The first guidelines for CEUS were introduced in 2004, with an initial focus on hepatic applications, particularly in the detection and characterization of focal liver lesions [2]. In 2008, the guidelines were expanded to include non-hepatic uses, reflecting the growing evidence supporting CEUS in a variety of clinical domains [3].

CEUS typically uses a microbubble UCA consisting of sulfur hexafluoride gas surrounded by a phospholipid shell, with microbubbles sized 3-5 μm. These microbubbles remain within the blood pool and cannot cross the endothelium, making them purely intravascular [4,5]. When exposed to an ultrasound beam, the microbubbles oscillate non-linearly, producing harmonic frequencies that are detected by the transducer. These harmonic frequencies allow the scanner to distinguish the UCA from static tissue, creating contrast-specific images through the pulse-inversion technique, which is similar to digital subtraction angiography and provides real-time images with high spatial and temporal resolution [6,7]. Once administered, UCAs are believed to pass through three distinct phases: the arterial phase (12-25 seconds post-injection), the portal or venous phase (30-80 seconds post-injection), and the late phase (beyond 90 seconds post-injection). Due to their specific properties, there is no parenchymal phase, and the contrast enhancement typically persists for around 8 minutes [8].

Safety profile

The metabolism and excretion of UCAs confer a favorable safety profile. The phospholipid shell is metabolized by the liver, and the sulfur hexafluoride gas is eliminated via exhalation through the lungs, bypassing renal excretion entirely [6]. This pharmacokinetic profile allows UCAs to be safely administered to patients with renal insufficiency, in contrast to iodinated and gadolinium-based agents used in Computed Tomography (CT) and Magnetic Resonance Imaging (MRI), which carry risks of nephrotoxicity and nephrogenic systemic fibrosis (NSF), respectively [9,10].

Because the microbubbles remain strictly intravascular, systemic toxicity is minimal [11]. Adverse events are rare and typically mild, with headache, nausea, or transient chest discomfort being the most frequently reported. Importantly, according to the European Federation of Societies for Ultrasound in Medicine and Biology (EFSUMB), UCAs have a lower incidence of anaphylactoid reactions compared with CT and MRI contrast agents (0.014% vs. 0.035-0.095% and 0.001-0.01%, respectively) [12]. This reinforces their favorable risk-benefit ratio in clinical practice.

Comparison of imaging modalities

The key differences between CEUS, CT, and MRI are summarized in Table 1. CEUS offers a unique combination of very low contrast risk, excellent renal safety, real-time imaging capability, and the absence of ionizing radiation, positioning it as a safe and efficient diagnostic option in many clinical scenarios [13].

**Table 1:** Comparison of CEUS, CT, and MRI CEUS: Contrast-Enhanced Ultrasound; CT: Computed Tomography; MRI: Magnetic Resonance Imaging; NSF: Nephrogenic Systemic Fibrosis [13]

Modality	Contrast Risk	Renal Safety	Cost	Real-Time Imaging	Ionizing Radiation
CEUS	Very Low	Safe	Low	Yes	No
CT	Moderate (iodine)	Risky	Moderate	No	Yes
MRI	Low (gadolinium)	Risk in NSF	High	No	No

## Review

Method

We undertook a non-systematic narrative review of the current literature on the clinical use of CEUS in Urology. A broad selection of recent publications, systematic reviews, meta-analyses, and guideline reports was screened in order to provide an updated overview of CEUS use in different urological diseases, such as kidney, bladder, testicular, prostatic, and fertility-associated conditions. The selection of studies was based on their clinical significance, relevance, and potential to contribute to the body of knowledge of CEUS rather than through a systematic, structured, protocol-based search.

Role in renal mass imaging

Advances in imaging technology have led to an increase in the detection of renal cell carcinoma (RCC), with approximately 60% of cases now identified incidentally during imaging studies [14]. A small solid renal mass (sSRM) refers to a renal tumor that is discovered incidentally on imaging and measures 4 cm or less in its largest dimension [15]. The widespread use of abdominal imaging for unrelated conditions, including ultrasound, CT, and MRI, has significantly contributed to the rising incidence of SRMs, many of which are clinically indolent. However, the biological heterogeneity of these lesions, ranging from benign entities such as oncocytomas and angiomyolipomas to malignant RCC subtypes, poses diagnostic challenges that influence treatment decisions, which can range from active surveillance to surgical resection. Therefore, precise characterization of these lesions is essential to avoid overtreatment and unnecessary interventions.

CEUS has emerged as a valuable imaging tool in this context. CEUS is quite effective in differentiating benign from malignant SRMs, providing real-time vascular assessment, and demonstrating high diagnostic reliability [16]. It offers dynamic evaluation of tumor vascularity, which can reflect underlying histopathological features such as angiogenesis, an important hallmark of malignancy. A study by Urraro et al evaluated the diagnostic efficacy of CEUS in distinguishing malignant from benign SRMs when conventional CT or MRI results are inconclusive, and demonstrated high diagnostic accuracy, with CEUS showing a sensitivity of 87.5%, specificity of 94%, positive predictive value of 95%, and negative predictive value of 86% [16].

A recent systematic review and meta-analysis [17] encompassing a total of 514 patients with SRMs demonstrated that CEUS exhibits high diagnostic accuracy, with a sensitivity of 94% and specificity of 78%, supporting its utility as a reliable imaging tool for renal mass evaluation. Importantly, CEUS can reduce the reliance on invasive diagnostic approaches such as biopsy, which, while informative, carries risks of bleeding, sampling error, and potential delay in definitive treatment.

CEUS also significantly enhances the characterization of complex renal cysts by delineating features such as septations, wall thickening, and nodular enhancement, which are essential for Bosniak classification and risk stratification. In a prospective study [18], CEUS demonstrated the highest diagnostic accuracy (80-83%) compared to CT (63-75%) and unenhanced sonography (30%) in detecting malignant features in complex cystic renal masses. These findings highlight CEUS as a superior modality in cases where accurate categorization of cystic lesions directly impacts clinical management, including decisions regarding surgical intervention versus conservative follow-up.

Another study evaluated the diagnostic performance of CEUS in characterizing indeterminate renal masses, analyzing data from 721 patients with 1,018 lesions, and comparing CEUS findings with pathology results and long-term follow-ups [19]. CEUS demonstrated high sensitivity (100%) and specificity (95%), offering an accurate method for distinguishing between benign and malignant masses. Beyond diagnostic accuracy, CEUS was shown to provide reliable lesion characterization that helped reduce unnecessary biopsies and surgeries, thereby contributing to patient safety and cost-effectiveness in renal mass management.

Taken together, these findings support the integration of CEUS into the routine evaluation of small renal masses, especially when CT/MRI results are equivocal. Its ability to provide dynamic, high-resolution vascular information in real-time positions CEUS as a critical adjunct in the diagnostic pathway, with significant implications for individualized patient management.

Role in bladder imaging

Cystoscopy remains the gold standard for bladder cancer diagnosis; however, CEUS enables detailed visualization of the bladder wall layers, helping to identify muscle invasion. After administration of contrast, the bladder wall appears as two distinct enhancing layers: the lamina propria and the muscularis propria [8]. If a tumor disrupts the continuity of the muscularis layer, it suggests muscle-invasive bladder cancer (MIBC), whereas preservation of the hypo-enhancing muscular layer indicates non-muscle-invasive bladder cancer (NMIBC) [20]. Accurate staging at the time of diagnosis is critical, as it directly guides therapeutic strategies: while NMIBC is typically managed with transurethral resection of bladder tumor (TURBT) and intravesical therapy, MIBC usually requires radical cystectomy, systemic therapy, or trimodal bladder-sparing approaches. Thus, a non-invasive, real-time modality capable of reliably distinguishing between NMIBC and MIBC is of considerable clinical importance.

A recent systematic review and meta-analysis (2023) evaluated the diagnostic performance of CEUS in staging bladder cancer, particularly its ability to differentiate MIBC from NMIBC preoperatively. The pooled analysis of five studies, involving 362 patients who underwent CEUS prior to TURBT, demonstrated high diagnostic accuracy, with both sensitivity and specificity at 88%, and an area under the summary receiver operating characteristic (SROC) curve of 0.94 [21]. These results highlight CEUS as a promising staging tool that could complement cystoscopy and cross-sectional imaging, particularly in resource-limited settings where MRI or CT may not be readily available.

CEUS is a highly accurate diagnostic and staging tool for bladder cancer, reaching levels of specificity and sensitivity in differentiating between Ta-T1 (low-stage) and T2 (muscle-invasive) bladder cancer, comparable to those demonstrated by reference standard methods [1]. Beyond tumor detection, CEUS provides functional insights into tumor perfusion and neovascularity, which are strongly associated with tumor aggressiveness. This vascular information may ultimately serve as a surrogate marker for tumor biology and prognosis.

A recent study involving 59 patients with bladder cancer reported that CEUS detected bladder lesions with an accuracy of 74.6%, which was only marginally lower than the 76.3% accuracy observed with MRI [22]. Notably, CEUS is more cost-effective and can be performed at the bedside, making it particularly useful for frail patients or those unfit for MRI due to comorbidities or contraindications such as pacemakers, severe claustrophobia, or renal impairment precluding gadolinium use.

A study by Gupta et al. further investigated the effectiveness of CEUS in assessing tumor grade and stage in 110 bladder cancer patients who underwent CEUS before endoscopic resection [23]. CEUS findings were compared with final histopathological outcomes. The analysis showed that CEUS identified NMIBC with 90% sensitivity and 75.71% specificity, and MIBC with 90.74% sensitivity and 92.76% specificity, confirming its high diagnostic reliability. Importantly, this suggests that CEUS can provide actionable information before definitive histology, assisting in surgical planning and patient counseling.

Taken together, these data suggest that CEUS could serve as a valuable adjunct in the diagnostic workup of bladder cancer, particularly in cases where MRI is unavailable or contraindicated. In addition to its diagnostic accuracy, CEUS offers several practical advantages: it avoids ionizing radiation, employs contrast agents with an excellent safety profile, and can be repeated during follow-up without cumulative risk. As a result, CEUS may not only complement cystoscopy for initial diagnosis and staging but also support longitudinal monitoring of treatment response or recurrence in selected patients.

Role in microdissection testicular sperm extraction (M-TESE)

Microdissection testicular sperm extraction (M-TESE) coupled with intracytoplasmic sperm injection (ICSI) has emerged as an effective treatment approach for individuals with non-obstructive azoospermia (NOA) [24]. However, sperm retrieval can be difficult under certain circumstances. The success rate of sperm retrieval in patients with NOA varies between 42% and 62%, and this is thought to be influenced by factors such as the retrieval technique, patient selection, and histological patterns [25]. NOA results from testicular failure, which can be due to genetic conditions (such as chromosomal abnormalities, translocations, and Y chromosome microdeletions), cryptorchidism, testicular torsion, radiation, or exposure to gonadotoxins. Despite the global impairment of spermatogenesis, it is well-established that small foci of active spermatogenic tissue can sometimes be present in the testes of men with NOA. Identifying and harvesting sperm from these foci is the cornerstone of successful M-TESE. Some studies have suggested a link between testicular blood flow and focal spermatogenesis [26].

M-TESE is an effective method for sperm extraction, using an operating microscope to identify larger, more opaque seminiferous tubules that are more likely to contain active spermatogenesis. This microsurgical approach minimizes unnecessary tissue damage and helps preserve the testicular blood supply, thereby reducing the risk of testicular atrophy or compromised endocrine function. However, sperm retrieval remains unsuccessful in a substantial proportion of patients with NOA because spermatogenesis is not uniformly distributed throughout the testis. Research has found that sperm quality and density are highest in areas with better blood flow, reinforcing the hypothesis that microvascular perfusion is closely correlated with focal spermatogenesis.

To improve retrieval outcomes, several imaging modalities have been explored. Techniques such as color Doppler ultrasound, power Doppler ultrasound, laser flowmetry, and magnetic resonance spectroscopy have been used to localize regions of spermatogenesis by assessing testicular perfusion. While these methods provide valuable information about the main vascular architecture, they are limited in their ability to visualize microvascular flow and capillary-level perfusion, which play a crucial role in supporting focal spermatogenesis [27,28]. This gap highlights the need for a more sensitive modality to guide surgical exploration.

CEUS, by visualizing microvascular blood flow, offers a unique advantage in this context. CEUS can detect subtle perfusion patterns within testicular parenchyma, enabling more precise identification of areas with a higher likelihood of spermatogenic activity. Integrating CEUS into M-TESE procedures could allow targeted sampling of optimally perfused regions, thereby improving sperm retrieval rates while reducing unnecessary dissection of non-productive tissue. In addition, CEUS may help minimize operative time and iatrogenic injury, ultimately preserving testicular function in men who may require repeat procedures.

Taken together, these insights suggest that CEUS has the potential to refine the surgical strategy for men with NOA undergoing M-TESE by improving the localization of spermatogenic foci and enhancing the efficiency and safety of sperm retrieval.

Role in scrotal imaging

CEUS is increasingly becoming indispensable in the acute scrotum workup. By providing real-time assessment of testicular perfusion and parenchymal integrity, it not only refines diagnosis in cases of trauma and tumor but also significantly impacts surgical decision-making. Its ability to distinguish viable from non-viable tissue enhances the likelihood of organ-preserving strategies, reduces unnecessary orchidectomies, and improves patient outcomes. CEUS, therefore, represents an essential adjunct to conventional ultrasound in both emergent and elective scrotal imaging.

Testicular Tumor

CEUS has emerged as a highly accurate tool for evaluating testicular tumors by assessing microvascular perfusion patterns that conventional ultrasound often misses. Malignant tumors typically demonstrate rapid wash-in/wash-out and heterogeneous enhancement, while benign or non-neoplastic lesions often lack internal enhancement or show only rim vascularity. These enhancement patterns can help differentiate seminomatous from non-seminomatous germ cell tumors, as the latter often display more heterogeneous enhancement due to necrosis and hemorrhage. Furthermore, CEUS has shown value in differentiating intratesticular neoplasms from mimicking conditions such as orchitis, granulomatous inflammation, or segmental infarction. By improving specificity, CEUS not only reduces unnecessary orchidectomies by confidently excluding benign lesions but also provides vascular patterns suggestive of specific tumor subtypes, supporting its role as a powerful adjunct to standard ultrasound in the diagnostic workup of testicular masses [29,30].

Testicular Rupture

Testicular rupture is an uncommon but serious condition that requires significant force from either blunt or penetrating trauma, leading to a breach in the integrity of the tunica albuginea. Conventional B-mode ultrasound can demonstrate heterogeneous echotexture, irregular testicular contours, or associated hematoceles, but may underestimate the true extent of parenchymal injury. CEUS provides clearer delineation of non-perfused, avascular areas corresponding to ischemia and necrosis, and more precisely visualizes the discontinuity of the tunica albuginea. This additional information is crucial for determining testicular salvageability and surgical planning. By allowing accurate distinction between salvageable and non-viable testicular tissue, CEUS supports timely testis-sparing surgery, which is particularly important in younger patients where preservation of fertility and endocrine function is a priority [31,32].

Testicular Fracture

Testicular fracture refers to a traumatic break in the testicular parenchyma, which may occur without a rupture of the tunica albuginea. On grayscale ultrasound, a testicular fracture can appear as hypoechoic linear bands or parenchymal heterogeneity, but its extent is often difficult to determine. CEUS enhances diagnostic accuracy by highlighting perfusion deficits, distinguishing avascular or hypovascular fracture planes from surrounding viable parenchyma. This information allows surgeons to accurately map the areas of preserved tissue, guiding more conservative interventions and avoiding unnecessary orchidectomy. As fertility preservation remains an important clinical consideration, CEUS provides critical insight into testicular viability in cases of trauma [33].

Traumatic Hematocele

A hematocele is a common scrotal injury resulting from blunt force trauma, where blood accumulates between the tunica vaginalis layers. Its sonographic appearance depends on the age of the collection: acute hematoceles appear echogenic due to fresh clot formation, whereas chronic hematoceles may appear complex with cystic degeneration, septations, and reduced volume [34]. Conventional ultrasound, however, often struggles to assess the tunica albuginea integrity in acute settings and may confuse hematocele with solid neoplasms or large intratesticular hematomas. CEUS improves diagnostic confidence by demonstrating the absence of internal vascularity within the collection, thereby differentiating it from neoplastic masses. It also provides a detailed assessment of testicular perfusion in adjacent parenchyma, ensuring that occult injuries such as fracture or rupture are not overlooked.

Role in prostate imaging

CEUS has emerged as a possible adjunctive tool in the detection and assessment of prostate cancer, leveraging its ability to image tumor-related neovascularity in real time. By improving microvascular blood flow imaging, CEUS enhances the visualization of suspicious lesions, particularly in the peripheral zone where most clinically significant tumors arise, and can guide targeted biopsies more accurately than standard systematic transrectal ultrasound (TRUS)-guided methods [35,36]. CEUS-guided targeting reduces the likelihood of missing clinically significant cancers while minimizing the detection of indolent tumors, thus supporting more precise risk stratification.

In a meta-analysis of 16 studies that included 2,624 patients, Li et al. reported that CEUS had a pooled sensitivity and specificity of 70% and 74%, respectively, for the detection of prostate cancer, demonstrating higher detection rates of clinically significant tumors when CEUS-targeted biopsies were coupled with systematic sampling [37]. Importantly, CEUS provides functional information on tumor vascularity, allowing distinction between more aggressive, hypervascular lesions and low-grade, less vascularized tumors. This capability positions CEUS as a potentially valuable tool not only in initial diagnosis but also in risk stratification.

CEUS offers a feasible alternative in patients unsuitable for multiparametric MRI (mpMRI)-for example, those with contraindications such as pacemakers, severe claustrophobia, or renal impairment limiting gadolinium use. In addition, CEUS has demonstrated utility in longitudinal disease monitoring, including patients under active surveillance protocols, where real-time vascular assessment may help detect early disease progression [38]. Furthermore, CEUS can also be applied after focal therapies (such as high-intensity focused ultrasound, cryotherapy, or focal laser ablation) to evaluate treatment response and confirm the absence of residual tumor vascularity.

While mpMRI remains the gold standard for prostate cancer imaging, CEUS offers a valuable complementary approach, particularly in scenarios where MRI access is limited, results are inconclusive, or patient-specific contraindications exist [39]. Beyond detection, CEUS enhances biopsy accuracy, improves the localization of clinically significant lesions, and supports individualized treatment planning by contributing to a more refined assessment of tumor biology. Given its relatively low cost, lack of ionizing radiation, and excellent safety profile of microbubble contrast agents, CEUS is increasingly recognized as a practical adjunct in the modern diagnostic algorithm for prostate cancer.

## Conclusions

CEUS is an increasingly valuable, non-invasive imaging modality with expanding applications in urology. Its real-time imaging capability, favorable safety profile, and ability to differentiate benign from malignant lesions enhance diagnostic accuracy, making it a useful adjunct to conventional imaging techniques such as CT and MRI. Despite its advantages, the widespread clinical adoption of CEUS remains limited due to restricted availability and the lack of fully established, standardized protocols across urological indications. Nonetheless, as standardization progresses and CEUS becomes more widely incorporated into diagnostic algorithms, it is poised to assume a more prominent role in routine urological practice.

## References

[1] Santarelli V, Rosati D, Canale V (2024). The current role of contrast-enhanced ultrasound (CEUS) in the diagnosis and staging of bladder cancer: A review of the available literature. Life.

[2] Nolsøe CP, Lorentzen T (2016). International guidelines for contrast-enhanced ultrasonography: Ultrasound imaging in the new millennium. Ultrasonography.

[3] Granata A, Campo I, Lentini P (2021). Role of contrast-enhanced ultrasound (CEUS) in native kidney pathology: Limits and fields of action. Diagnostics (Basel).

[4] Greis C (2004). Technology Overview: SonoVue (Bracco, Milan). Eur Radiol.

[5] Rafailidis V, Huang DY, Yusuf GT, Sidhu PS (2020). General principles and overview of vascular contrast-enhanced ultrasonography. Ultrasonography.

[6] Yusuf GT, Rafailidis V, Moore S (2020). The role of contrast-enhanced ultrasound (CEUS) in the evaluation of scrotal trauma: A review. Insights Imaging.

[7] Sridharan A, Eisenbrey JR, Forsberg F, Lorenz N, Steffgen L, Ntoulia A (2021). Ultrasound contrast agents: Microbubbles made simple for the pediatric radiologist. Pediatr Radiol.

[8] Srivastava S, Roekel DV, Wright JL, Bruce M, Dighe M (2023). Contrast-enhanced ultrasound (CEUS) in the evaluation of bladder pathologies: Review. WFUMB Ultrasound Open.

[9] Girometti R, Stocca T, Serena E, Granata A, Bertolotto M (2017). Impact of contrast-enhanced ultrasound in patients with renal function impairment. World J Radiol.

[10] Chang EH (2018). An introduction to contrast-enhanced ultrasound for nephrologists. Nephron.

[11] Moestue SA, Gribbestad IS, Hansen R (2012). Intravascular targets for molecular contrast-enhanced ultrasound imaging. Int J Mol Sci.

[12] Sidhu PS, Cantisani V, Dietrich CF (2018). The EFSUMB Guidelines and Recommendations for the Clinical Practice of Contrast-Enhanced Ultrasound (CEUS) in non-hepatic applications: Update 2017 (Long Version). Ultraschall Med.

[13] Lund M, Nadarevic T, Bjerre TA, Grønbæk H, Mortensen F, Kragh Andersen P (2020). Contrast‐enhanced ultrasound compared with computed tomography, magnetic resonance imaging, and positron emission tomography‐computed tomography for diagnosing liver metastases in people with newly diagnosed colorectal cancer. Cochrane Database Syst Rev.

[14] Elbanna KY, Jang HJ, Kim TK, Khalili K, Guimarães LS, Atri M (2021). The added value of contrast-enhanced ultrasound in evaluation of indeterminate small solid renal masses and risk stratification of cystic renal lesions. Eur Radiol.

[15] Gordetsky J, Eich ML, Garapati M, Del Carmen Rodriguez Pena M, Rais-Bahrami S (2019). Active Surveillance of Small Renal Masses. Urology.

[16] Urraro F, Piscopo M, Giordano N (2024). Diagnostic value of contrast-enhanced ultrasound in differentiating malignant from benign small renal masses after CT/MRI. J Clin Med.

[17] Tufano A, Antonelli L, Di Pierro GB (2022). Diagnostic performance of contrast-enhanced ultrasound in the evaluation of small renal masses: A systematic review and meta-analysis. Diagnostics.

[18] Quaia E, Bertolotto M, Cioffi V, Rossi A, Baratella E, Pizzolato R, Cov MA (2008). Comparison of contrast-enhanced sonography with unenhanced sonography and contrast-enhanced CT in the diagnosis of malignancy in complex cystic renal masses. AJR Am J Roentgenol.

[19] Barr RG, Peterson C, Hindi A (2013). Evaluation of indeterminate renal masses with contrast-enhanced US: A diagnostic performance study. Radiology.

[20] Caruso G, Salvaggio G, Campisi A, Melloni D, Midiri M, Bertolotto M, Lagalla R (2010). Bladder tumor staging: Comparison of contrast-enhanced and gray-scale ultrasound. AJR Am J Roentgenol.

[21] Tufano A, Rosati D, Moriconi M (2024). Diagnostic accuracy of contrast-enhanced ultrasound (CEUS) in the detection of muscle-invasive bladder cancer: A systematic review and diagnostic meta-analysis. Curr Oncol.

[22] Li C, Gu Z, Ni P (2021). The value of contrast-enhanced ultrasound and magnetic resonance imaging in the diagnosis of bladder cancer. J Cancer Res Ther.

[23] Gupta VG, Kumar S, Singh SK, Lal A, Kakkar N (2016). Contrast enhanced ultrasound in urothelial carcinoma of urinary bladder: An underutilized staging and grading modality. Cent European J Urol.

[24] Zhang S, Du J, Tian R, Xie S, Li F, Li Z (2018). Assessment of the use of contrast enhanced ultrasound in guiding microdissection testicular sperm extraction in nonobstructive azoospermia. BMC Urol.

[25] Dabaja AA, Schlegel PN (2013). Microdissection testicular sperm extraction: An update. Asian J Androl.

[26] Raman JD, Schlegel PN (2003). Testicular sperm extraction with intracytoplasmic sperm injection is successful for the treatment of nonobstructive azoospermia associated with cryptorchidism. J Urol.

[27] Herwig R, Tosun K, Pinggera GM (2004). Tissue perfusion essential for spermatogenesis and outcome of testicular sperm extraction (TESE) for assisted reproduction. J Assist Reprod Genet.

[28] Souza CA, Cunha-Filho JS, Fagundes P, Freitas FM, Passos EP (2005). Sperm recovery prediction in azoospermic patients using Doppler ultrasonography. Int Urol Nephrol.

[29] Yu X, Zhao Q, Guo L (2024). Diagnostic performance of contrast-enhanced ultrasound in testicular space-occupying lesions: A systematic review and meta-analysis. Acad Radiol.

[30] Isidori AM, Pozza C, Gianfrilli D (2014). Differential diagnosis of nonpalpable testicular lesions: Qualitative and quantitative contrast-enhanced US of benign and malignant testicular tumors. Radiology.

[31] Valentino M, Bertolotto M, Derchi L, Bertaccini A, Pavlica P, Martorana G, Barozzi L (2011). Role of contrast enhanced ultrasound in acute scrotal diseases. Eur Radiol.

[32] Hedayati V, Sellars ME, Sharma DM, Sidhu PS (2012). Contrast-enhanced ultrasound in testicular trauma: Role in directing exploration, debridement and organ salvage. Br J Radiol.

[33] Missiroli C, Mansouri M, Singh A (2024). Imaging of acute conditions of male reproductive organs. Emergency Radiology: Imaging of Acute Pathologies.

[34] Deurdulian C, Mittelstaedt CA, Chong WK, Fielding JR (2007). US of acute scrotal trauma: Optimal technique, imaging findings, and management. Radiographics.

[35] Smeenge M, Mischi M, Laguna Pes MP, de la Rosette JJ, Wijkstra H (2011). Novel contrast-enhanced ultrasound imaging in prostate cancer. World J Urol.

[36] Liu Y, Zeng S, Xu R (2022). Application of multiple ultrasonic techniques in the diagnosis of prostate cancer. Front Oncol.

[37] Li Y, Tang J, Fei X, Gao Y (2013). Diagnostic performance of contrast enhanced ultrasound in patients with prostate cancer: A meta-analysis. Acad Radiol.

[38] Lee CH, Tan TW, Tan CH (2021). Multiparametric MRI in active surveillance of prostate cancer: An overview and a practical approach. Korean J Radiol.

[39] Liu Y, Zeng S, Zhou D (2025). Diagnostic performance of multiple ultrasonic modalities for prostate cancer. Clinics.

